# Acute Flaccid Paralysis with Anterior Myelitis — California, June 2012–June 2014

**Published:** 2014-10-10

**Authors:** Patrick Ayscue, Keith Van Haren, Heather Sheriff, Emmanuelle Waubant, Paul Waldron, Shigeo Yagi, Cynthia Yen, Anna Clayton, Tasha Padilla, Chao Pan, John Reichel, Kathleen Harriman, James Watt, James Sejvar, William Allan Nix, Daniel Feikin, Carol Glaser

**Affiliations:** 1 Epidemic Intelligence Service, CDC; 2 California Department of Public Health; 3 Stanford University, Stanford, California; 4 Lucile Packard Children’s Hospital, Palo Alto, California; 5 University of California San Francisco Multiple Sclerosis Center, San Francisco, California; 6 National Center for Zoonotic, Vectorborne, and Enteric Diseases, CDC; 7 National Center for Immunization and Respiratory Diseases, CDC

In August 2012, the California Department of Public Health (CDPH) was contacted by a San Francisco Bay area clinician who requested poliovirus testing for an unvaccinated man aged 29 years with acute flaccid paralysis (AFP) associated with anterior myelitis (i.e., evidence of inflammation of the spinal cord involving the grey matter including anterior horn cell bodies) and no history of international travel during the month before symptom onset. Within 2 weeks, CDPH had received reports of two additional cases of AFP with anterior myelitis of unknown etiology. Testing at CDPH’s Viral and Rickettsial Disease Laboratory for stool, nasopharyngeal swab, and cerebrospinal fluid (CSF) did not detect the presence of an enterovirus (EV), the genus of the family *Picornaviridae* that includes poliovirus. Additional laboratory testing for infectious diseases conducted at the CDPH Viral and Rickettsial Disease Laboratory did not identify a causative agent to explain the observed clinical syndrome reported among the patients. To identify other cases of AFP with anterior myelitis and elucidate possible common etiologies, CDPH posted alerts in official communications for California local health departments during December 2012, July 2013, and February 2014. Reports of cases of neurologic illness received by CDPH were investigated throughout this period, and clinicians were encouraged to submit clinical samples for testing. A total of 23 cases of AFP with anterior myelitis of unknown etiology were identified. Epidemiologic and laboratory investigation did not identify poliovirus infection as a possible cause for the observed cases. No common etiology was identified to explain the reported cases, although EV-D68 was identified in upper respiratory tract specimens of two patients. EV infection, including poliovirus infection, should be considered in the differential diagnosis in cases of AFP with anterior myelitis and testing performed per CDC guidelines (1).

A case was defined as AFP in at least one limb consistent with anterior myelitis, as indicated by neuroimaging of the spine or electrodiagnostic studies (e.g., nerve conduction studies and electromyography), and with no known alternative etiology, in a person with symptom onset during January 2012–June 2014. Among the 23 cases identified, younger persons and males were more frequently affected, with a median age of 10 years (range = 1–73 years); 15 were aged <15 years, and 56% were male. Similar to the race/ethnicity distribution in California, seven (30%) patients were white, six (26%) were Asian, six (26%) were Hispanic, one (4%) was black, one (4%) was of multiple race, and two (9%) were of unknown race. Affected patients resided in diverse geographic areas throughout California with no indication of clustering. During the 30-month inquiry, no indication of seasonality or temporal trends in disease onset was established (Figure).

Common features among the clinical presentations of patients included an upper respiratory or gastrointestinal prodrome <10 days before AFP onset (83%), CSF pleocytosis (83%), and absence of sensory deficits (78%). Ten patients (43%) also had concomitant mental status changes; eight patients (34%) had cranial nerve abnormalities. Patients typically had extended hospital stays (median = 17 days), and of 13 patients with available information, all had prolonged paralysis persisting at 60 days follow-up. Five patients were ventilator-dependent when discharged from the hospital to rehabilitation facilities, and one death was reported in an adult. Of 10 patients with mental status changes, eight (80%) had returned to baseline cognitive function at the time of discharge.

Specimens were available for testing from 19 (83%) of the patients. The CDPH Viral and Rickettsial Disease Laboratory tested nasopharyngeal or throat swabs (18), stool or rectal swabs (14), serum (17), and CSF (17) for evidence of recent infection with numerous infectious agents, including EVs (including poliovirus), arboviruses, herpes viruses (HSV-1, HSV-2, VZV, and EBV), parechoviruses, adenoviruses, rabies, influenza A and B, human metapneumovirus, respiratory syncytial virus, parainfluenza 1–4, *Mycoplasma pneumoniae*, rickettsial pathogens, and free-living amoebas. Results were unremarkable except for the following: 1) mycoplasma immunoglobulin M–positive serologies in two patients (these same patients had negative mycoplasma throat polymerase chain reaction [PCR] tests), 2) rhinovirus-positive PCR from a respiratory specimen in one patient, and 3) EV-positive PCR from throat or upper respiratory tract specimens in two patients; these EVs were sequenced as EV-D68. Testing was limited by incomplete and late collection of specimens; respiratory samples collected <7 days of paralysis onset were submitted for nine (39%) patients, and stool or rectal specimens were submitted for 15 (65%) patients. Specimens meeting World Health Organization (WHO) or CDC guidelines for poliovirus detection (e.g., two stool specimens collected ≥24 hours apart and <14 days after symptom onset) were submitted for only two of the patients. Serologic testing was of limited value because specimens often were collected after patients had received intravenous immunoglobulin (IVIG) therapy.

Poliovirus was determined to be an unlikely etiology for any of the cases based on epidemiologic and limited laboratory investigation findings. Nonetheless, because AFP with anterior myelitis is the classic presentation of paralytic poliomyelitis, CDPH attempted to rule out poliovirus infection. Of 14 patients with available information, 12 had previously received polio vaccine; one child and one adult were unvaccinated because of personal belief exemptions. Pre-IVIG serum was available from the unvaccinated child and tested negative for neutralizing antibodies against poliovirus at CDC laboratories. None of the patients reported travel out of the United States during the month before symptom onset.

## Discussion

AFP has numerous etiologies and can prove diagnostically challenging; however, after the widespread implementation of polio vaccination worldwide, the subset of patients suffering from AFP with anterior myelitis is markedly smaller than the population of patients suffering from other forms of AFP. AFP is not a reportable syndrome in California, or any other U.S. state, other than as an occurrence of an unusual disease, and whether these cases represent an actual increase from baseline incidence of AFP with anterior myelitis in this population is unclear. A study examining AFP in children aged <15 years in California during 1992–1998 reported an incidence of 1.4 AFP cases per 100,000 children per year, with the most common diagnoses being Guillain-Barré syndrome (23%), unspecified AFP (21%), and botulism (12%). None of the 245 reviewed cases had recognized anterior myelitis, which is characteristic of paralytic poliomyelitis (2).

The etiology of AFP with anterior myelitis in the cases described in this report remains undetermined. EVs circulate widely in the United States, causing an estimated 10–15 million symptomatic, mostly nonneurologic illnesses annually (2,3). EVs, other than poliovirus, are rarely known to result in AFP with anterior myelitis. EV-D68 infections in most patients manifest as purely respiratory illnesses. A single case of an EV-D68 infection associated with AFP has been reported in the literature (4), and an additional case was reported through nationwide EV surveillance (5). CDC is working with state and local health departments to better characterize the respiratory disease health burden and spectrum of illness associated with the recent increase in EV-D68 respiratory illness across the United States (6). The significance of EV-D68 detection in two of the cases in this report is unclear, particularly because it was detected in upper respiratory tract specimens and not CSF. However, EVs can prove challenging to identify as a cause of neurologic syndromes, including AFP. Poliovirus and EV-A71, well-documented causes of serious neurologic disease including poliomyelitis-like paralysis, are infrequently recovered from spinal fluid (7,8). In addition, delayed collection of laboratory specimens after respiratory illness and paralysis onset might have reduced the capacity to recover etiologic agents.

Paralysis caused by poliovirus infection results from anterior horn cell injury and is characterized by poor recovery of motor function. Sensory loss, as reported in 22% of the cases in this report, is not a feature commonly associated with patients with paralysis because of poliovirus infection. However, sensory symptoms (e.g., pain and paresthesia) have been reported with poliovirus infections. The last cases of paralytic poliomyelitis caused by endemic transmission of wild poliovirus in the United States occurred in 1979. Global poliovirus eradication efforts have greatly reduced the risk for introduction of poliovirus into the United States; wild-type poliovirus is currently endemic only in Afghanistan, Nigeria, and Pakistan; however, polio has been exported to countries that have previously been polio-free, and seven other countries have had cases or transmission of wild poliovirus in the last 12 months (9). Cases of vaccine-associated paralytic poliomyelitis cases do occur in countries using oral poliovirus vaccine (OPV) (10). OPV is no longer available in the United States; inactivated poliovirus vaccine has been recommended exclusively in the United States since 2000.

Although polio is no longer endemic in the United States, ruling out poliovirus infection in clinically compatible, unexplained cases of AFP, particularly those with anterior myelitis, is important to ensure that any importation of poliovirus is quickly identified and investigated. WHO and CDC have guidelines for epidemiologic, clinical, and laboratory investigations of AFP to rule out poliovirus infection (10). Clinical and epidemiologic investigation should include a careful neurologic examination to characterize specific sensory (e.g., sensory symptoms versus sensory loss) as well as motor findings, querying patients about recent international travel (<30 days before onset), and contact with persons who recently traveled, particularly to regions with polio cases or regions where OPV is used. Documented history of vaccination and whether inactivated poliovirus vaccine or OPV was administered should be noted, including dates of administration, number of doses, and manufacturer, if the information is available.

Specimens should be collected early during the course of disease for laboratory testing. Collection of specimens should follow CDC and WHO guidelines and include two stool specimens collected ≥24 hours apart and <14 days after symptom onset, serum before administration of IVIG, and throat swabs. Of patients who had samples tested at the CDPH Viral and Rickettsial Disease Laboratory as described in this report, only two met the specifications for ruling out poliovirus infection as recommended by WHO or CDC guidelines*†§; all others lacked two stool specimens collected ≥24 hours apart and <14 days after symptom onset.

Paralytic poliomyelitis cases are immediately reportable to all state and local health departments in the United States. A confirmed paralytic poliomyelitis case should be reported to CDC within 4 hours after meeting notification criteria.

Although AFP with anterior myelitis or grey matter involvement comprises a subset of patients with AFP, these cases can be challenging to distinguish at initial presentation before clinical, imaging, and laboratory study results are available. Thus, specimen collection to definitively rule out poliovirus infection from possible differential diagnoses should be considered among all patients with AFP of unknown etiology or a suspected viral etiology. This is particularly important for persons who are unimmunized and have a history of travel to countries with endemic polio or countries that use OPV for routine immunization.

Physicians treating patients with AFP of unknown etiology should work with their local and state health departments to rule out poliomyelitis early during the course of disease. To ensure adequate specimens for poliovirus testing, specimens should be collected according to CDC and WHO guidelines.

What is already known on the topic?

Acute flaccid paralysis (AFP) with anterior myelitis is not a reportable condition, and baseline rates of disease are unknown but are likely quite low. Data from 1992–1998 on children aged <15 years in California indicated an incidence of 1.4 AFP cases per 100,000 children per year and did not identify a single case of AFP with anterior myelitis. Viral causes of AFP with anterior myelitis include enteroviruses (including poliovirus), adenovirus, and flaviviruses such as West Nile virus. Enterovirus D68 has previously been reported to be associated with neurologic illness, although the scope of neurologic manifestations is unclear.

What is added by the report?

A total of 23 cases of AFP with anterior myelitis were identified during 2012–2014 in California. No common etiology was identified, although clinical laboratory findings supported a viral etiology. Two patients tested positive for enterovirus D68 from upper respiratory specimens.

What are the implications for public health practice?

Poliovirus infection should be ruled out in all cases of AFP with anterior myelitis of unknown etiology. The scope of illness associated with enterovirus D68 might include neurologic manifestations, including AFP.

## Figures and Tables

**FIGURE:**
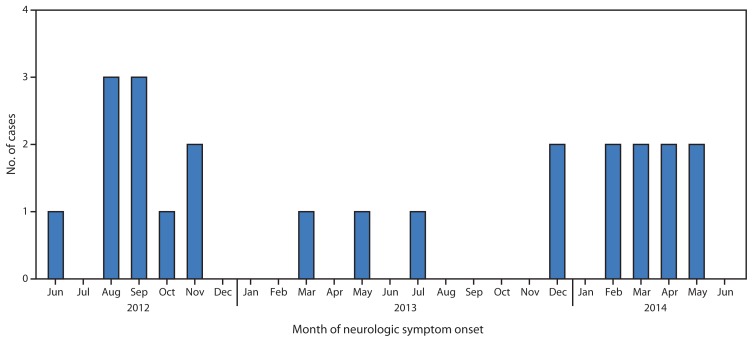
Number of cases of acute flaccid paralysis with anterior myelitis (N = 23), by month of neurologic symptom onset — California, 2012–2014

## Footnotes

* Additional information about CDC guidelines is available at http://www.cdc.gov/vaccines/pubs/surv-manual/chpt12-polio.html.

† Additional information about WHO guidelines are available at https://www.hpsc.ie/hpsc/a-z/vaccinepreventable/polio/guidance/file,2461,en.pdf.

§ Additional information about CDPH specimen collection guidelines for infectious disease testing in cases of neurologic illness is available at http://www.cdph.ca.gov/programs/vrdl/pages/neurologicsurveillancetesting.aspx.
